# Analysis of infrared radiation emitted by moxibustion devices made of different materials using Fourier transform infrared spectroscopy

**DOI:** 10.1016/j.heliyon.2024.e33221

**Published:** 2024-06-18

**Authors:** Jiachen Zhang, Jing He, Shuang Shuang, Yuqing Shi, Li Han, Xin Hui, Xiali Ouyang, Jingyi Zhu, Zhongyu Wang, Baixiao Zhao, Rui He

**Affiliations:** a School of Acupuncture, Moxibustion and Tuina, Beijing University of Chinese Medicine, Beijing, PR China; b National Institute of Clean-and-Low-Carbon Energy, Beijing, PR China; c School of Traditional Chinese Medicine, Beijing University of Chinese Medicine, Beijing, PR China; d Beijing Aerospace General Hospital, Beijing, PR China; e School of Life Sciences, Beijing University of Chinese Medicine, Beijing, PR China

**Keywords:** Traditional Chinese medicine, Integrative medicine, Moxibustion, Fourier transform infrared spectroscopy, Combustion stability, Infrared radiation intensity, Principal component analysis

## Abstract

Moxibustion has a long history of use as a traditional Chinese medicine therapy. Infrared radiation is an important and effective factor in moxibustion. Instead of the time-consuming and laborious process of holding moxa sticks in the hand, moxibustion devices are commonly used as moxibustion methods and tools in modern times. With the publication of the international standard of moxibustion devices (ISO18666:2021, Traditional Chinese Medicine - General requirements of moxibustion devices) published, moxibustion devices of various materials are now sold in the pharmacies and online stores. However, the influence of moxibustion devices on the therapeutic effect of moxibustion has not been studied. Therefore, this research was aimed to evaluate the infrared radiation of moxibustion devices, in order to select the moxibustion device that delivered infrared radiation closest to that of moxa stick combustion. The combination of combustion stability and infrared radiation intensity showed that cardboard tubes and silicone were better materials for moxibustion devices. In the mid-far infrared wave band, the moxibustion devices made from cardboard tubes and silica gels can better maintain the thermal effect generated by moxibustion and enable it to be more easily absorbed by the human body. The infrared radiation intensity of the cardboard moxibustion devices increased rapidly and steadily and could be maintained for the longest time. In conclusion, cardboard tubes are the better material for moxibustion devices with respect to infrared radiation.

## 1 Introduction

Moxibustion is an important external therapy in traditional Chinese medicine (TCM) [1,2]. The infrared light, heat, and smoke generated by igniting moxibustion materials have a therapeutic effect on the human body in preventing diseases or modifying disease status [3,4]. It has been reported that moxibustion has the effects of reducing inflammation, preventing mild cognitive impairment, improving reproductive function, etc [[5], [6], [7]]. From ancient times to the present day, moxibustion has been used by igniting moxa sticks rolled from mugwort for acupoints. The effective elements of moxibustion include heat, infrared radiation, and moxa smoke [8]. In addition to the medicinal and thermal features of moxibustion, the infrared radiation of moxibustion is also an important factor [[9], [10], [11]]. The infrared generated by the burning of moxa sticks can provide energy for the body's metabolic and immune functions [12]. In the past, moxibustion for patients usually used moxa cones or holding moxa sticks by traditional Chinese doctors [13]. In the past, traditional Chinese doctors usually treated patients using moxa cones or by holding moxa sticks. Nowadays, moxibustion devices are widely used. Doctors put the ignited moxa stick into the moxibustion device and place it on the acupoint, which makes the clinical use of moxibustion easy and fast.

With more widespread use of moxibustion devices, the International Organization for Standardization published the international standard for moxibustion devices (ISO18666:2021, Traditional Chinese medicine - General requirements of moxibustion devices). Moxibustion devices can be used to hold the moxa sticks firmly and to adjust temperature. The size and shape should be suitable to cover on a specific acupoint or an area of the human body surface. A safety arrangement is necessary to prevent ash or ember from falling onto the surface of the human body. However, the international standard places no restrictions on the materials used for making moxibustion devices. , although the different materials used for moxibustion devices influence the infrared radiation spectrum from combustion of moxa sticks [14].

The body of moxibustion devices are usually made from woods, ceramics, cardboard tubes, silica gels, etc. The strength of infrared radiation is closely related to the differences in production materials. Any object with a temperature above absolute zero can release energy. The invisible infrared light generated during the combustion of moxa sticks accounts for the majority of their radiation spectrum [15], which varies in intensity from red light to far infrared, and it is mainly in the near infrared wave band. Current research indicates that near-infrared and far-infrared have positive effects on the human body [[16], [17], [18], [19]]. The different materials used for moxibustion devices can cause affect the changes in radiation intensity [20].

The aim of our study was to observe the Fourier transformation infrared spectroscopy of four types of moxibustion devices, including cardboard tubes, silica gels, woods, and ceramics. We compared the advantages and disadvantages of four types of moxibustion devices with respect to combustion stability and infrared radiation for different times and wavebands. Our results can provide the support and basis for the international standardization of moxibustion devices.

## 2 Experimental

### 2.1 Measurement of FTIR spectra

**Fig. 1:**
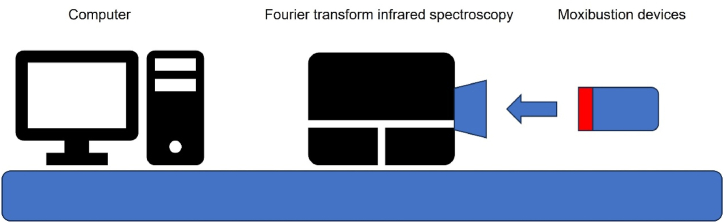
Experimental platform layout diagram.

In this experiment, we chose the Nicolet iS50 Fourier infrared transform spectrometer (Thermo Fisher, USA) to detect the spectra of the moxibustion devices (see Fig. 1). Each spectrum was complied from the average of 32 scans in the range of 1.28 μm–25μm. The ambient temperature of the experiment was 22 °C ± 3 °C, the relative humidity was 55 % ± 10 %, and the vibration source around the instrument was avoided while the spectrum was collected. Before the experiment, all the moxibustion devices were placed in a constant environment (the same temperature and humidity conditions) for 72 h to ensure the uniformity of the moisture content of the samples.

The specific spectrum collection process was as follows:

First, the background radiation spectrum was collected after a 20 min’ stabilization of the spectrometer. Because water or carbon dioxide in the air would absorb the infrared light of moxibustion devices, eliminating these influences provides greater accuracy.

Secondly, moxa sticks were ignited and put into each type of moxibustion device. We wet the cowhide with water around 37 °C which aimed to simulate the temperature of human skin and put it under each device. Moxibustion devices were placed horizontally about 8 cm in front of the detection window, and we recorded this moment as the 0 min in the combustion process of moxibustion combustion.

Finally, the spectrum test was carried out at the corresponding time point. We wet the cowhide at the end of each spectrum collection and then saved the spectrum for follow-up analysis. Each moxibustion device was tested in turn to prevent temperature of the moxibustion device from rising all the time because of the continuous testing.

### 2.2 Materials of moxibustion devices

**Fig. 2:**
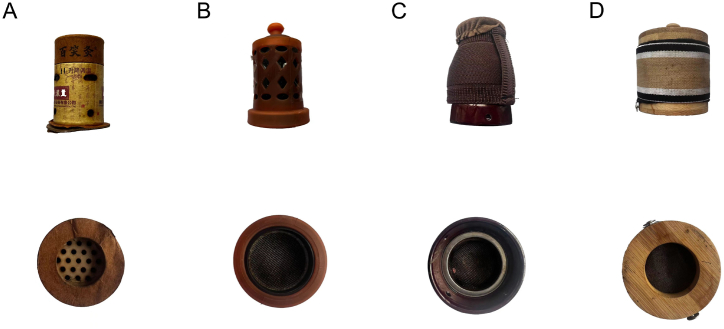
Appearance of four types of moxibustion devices A) Cardboard moxibustion devices, B) Silicone moxibustion devices, C) Ceramic moxibustion devices D) Wooden moxibustion devices.

We used moxa sticks made by Chongqing Baixiao Co., LTD for all the moxibustion devices to eliminate any impact on the spectrum of moxa sticks produced by different manufacturers. Cardboard moxibustion devices (MD) were purchased from Chongqing Baixiao Co., LTD. Wooden moxibustion devices were purchased from Qichun Chutian Yangshengtang Qidai products Co., LTD. Ceramic moxibustion devices were purchased from Changsha Yaofei Network Technology Co., LTD. Silicone moxibustion devices were purchased from the Qi moxibustion factory outlet store. The four types of moxibustion devices are shown in Fig. 2.

We collected the infrared radiation spectrum of the human body after treatment by moxibustion. The construction of the bottom of the moxibustion device was usually designed to be poriferous, and the light detector of the FTIR spectrometer was able to go through the holes and measure the spectra of the moxa sticks inside the moxibustion devices. We decided to use a cushion of isolation material as the simulated skin beneath the bottom of the moxibustion devices, and we selected cowhide, filter paper, and biological semi permeable membrane (SPM) as the isolation materials. To compare with the infrared radiation spectrum of the human body, we chose the best isolation material to simulate human skin.

### 2.3 Measurement and analysis of FTIR spectra at different time points

Generally speaking, the therapeutic process of moxibustion would take 20 min in a TCM clinic. In order to measure the variation of infrared radiation of the moxibustion devices, the infrared spectra were detected at the 1st, 3rd, 5th, 7th, 9th, 11th, 13th, 15th, 17th, and 19th minutes after the moxa sticks were ignited in the moxibustion devices. We then utilized the repeat analysis to explore the intensity of the devices at different time points. The intensity of 1.28 μm–25 μm at every time point was measured and analyzed.

### 2.4 Measurement of combustion stability

In this experiment, the stability of the infrared radiation of the moxibustion devices was measured by the relative standard deviation. We detected 10 infrared spectra of moxibustion devices at the 1st, 3rd, 5th, 7th, 9th, 11th, 13th, 15th, 17th, 19th minutes and calculated the standard deviation using OMNIC software. Finally, we saved the standard deviation spectra, and the average value of the standard deviation graph was recorded as the relative standard deviation value of the corresponding moxibustion devices: the greater the standard deviation, the smaller the stability of the infrared radiation of the moxibustion devices.

### 2.5 Analysis methods for different wavebands

Four wavebands were analyzed, including the detection band of the FTIR spectrometer (1.28 μm–25μm), near-infrared radiation (1.28μm–2.5 μm), mid/far-infrared radiation (2.5 μm–25μm) and the peak band of human infrared radiation (7.5 μm–10 μm). Infrared radiation intensity at different wavebands were calculated by the algorithm ‘mean value measurement’ of the OMNIC software. The average infrared spectrum of each moxibustion device was calculated from the 10 spectra within 20 min by the algorithm ‘statistical spectrum and mean spectral calculation’. We calculated and recorded the respective mean value of infrared radiation intensity of the four wavebands by the algorithm ‘mean value measurement’.

### 2.6 Principal component analysis

Principal component analysis (PCA) of the infrared radiation spectra was performed by SPSS 19.0 and OriginPro 2021(OriginLab, USA). Firstly, the intensity of every moxibustion device at each time point were used as the components of the principal analysis. Two eigenvalue of the components which were greater than 1 were chosen as the two principal components, and each moxibustion device's score was calculated by eigenvalues. We also analyzed and compared the radiation intensity of the cardboard moxibustion devices with other moxibustion devices for the four different bands, in order to judge whether the intensity at different time points shows the same effect in different moxibustion devices.

### 2.7 Statistical analysis

The spectrometer was controlled by the software OMNIC (Thermo Fisher, USA), which could optimize the spectrum. The software OriginPro 2021 (OriginLab, USA) and GraphPad Prism9.0 (GraphPad Software, USA) was used to performed PCA and make the statistical graphs. One-way ANOVA with LSD test was used to test differences in each indicator between groups using SPSS 19.0 statistical software.

## 3 Results

### 3.1 FTIR spectral features of moxibustion devices

**Fig. 3:**
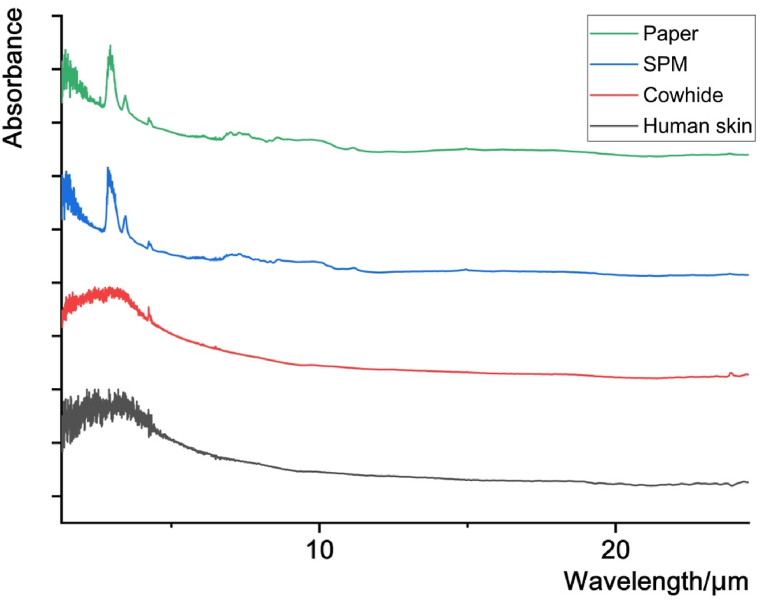
The FTIR spectra of the moxibustion device with paper, semi permeable membrane (SPM), cowhide and human skin.

Since infrared radiation passing through human skin produces a certain degree of attenuation [21], we put three different materials under the moxibustion device separately to simulate moxibustion on human skin. We chose paper, semi permeable membrane and cowhide as the barrier mediums. Fig. 3 shows the FTIR spectra of the moxibustion device for paper, semi permeable membrane and cowhide respectively, and the spectrum of the moxibustion applied to the human skin by a moxibustion device. The spectral shape of paper and semi permeable membrane is different from that of the human skin, and the two materials are not suitable for imitating moxibustion on the human skin. As the spectral shape of cowhide is more similar to that of the human skin, we selected the cowhide for the next experiment.

**Fig. 4:**
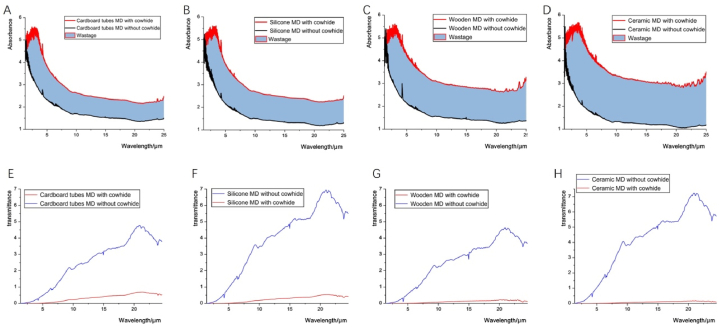
The FTIR spectra of the four types of moxibustion device with and without cowhide.

Fig. 4A–D shows the FTIR spectra of the four types of moxibustion device, with and without cowhide: the higher the absorbance, the lower the infrared radiation intensity of the moxibustion device. The shape of combustion infrared spectroscopy is regular and easy to analyze using absorbance as the vertical coordinate. Ceramic moxibustion devices without cowhide emit strong infrared radiation directly. The infrared radiation intensity of the moxibustion device used with wet cowhide is evidently reduced. The area of the blue region represents the wastage of infrared radiation passing through the cowhide. The ceramic moxibustion devices have the highest wastage of infrared radiation when passing through cowhide. The infrared radiation loss of cardboard moxibustion devices passing through cowhide is minimal. The cowhide absorbs a portion of infrared radiation, which is similar to moxibustion on the human body. The absorbance of cardboard and silica gel moxibustion devices by cowhide is significantly lower, which indicates that the two moxibustion devices release more energy. Wooden and ceramic moxibustion devices emit only weak infrared energy.

Fig. 4E–H shows the transmittance of the four types of moxibustion device, with and without cowhide. Transmittance (T) is inversely proportional to absorbance (A), and the relationship between A and T follows the formula. The four types of moxibustion devices have high transmittance of around 20 μm in wavelength. Ceramic and silicone moxibustion devices without cowhide reflect higher transmittance than the cardboard moxibustion devices. The cowhide greatly reduces the transmittance of infrared radiation of the moxibustion device. The absorbance of cardboard moxibustion devices with cowhide is obviously lower than those of the other three types of moxibustion devices. But the transmittance of cardboard moxibustion devices with cowhide is higher than the other moxibustion devices with cowhide (Fig. 4E-H, red line). In terms of transmittance, the cardboard moxibustion devices have good infrared radiation penetration power.

### 3.2 Combustion stability of moxibustion devices

In the test of combustion stability measurement, we used standard deviation of absorbance as a criterion for evaluating stability. The moxibustion devices made from the four different materials were measured 10 times separately and repeatedly. The standard deviation of absorbance is inversely proportional to the combustion stability: the smaller the standard deviation, the more stable the combustion stability of the moxa sticks with this device.

**Fig. 5:**
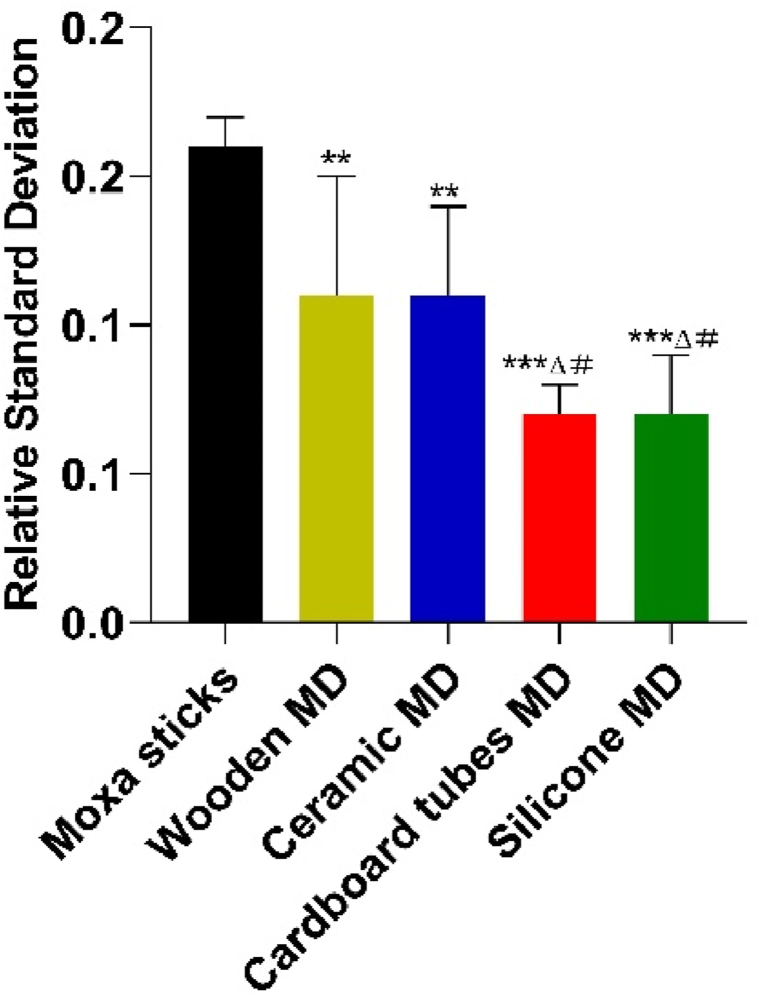
Combustion stability of moxibustion devices.

Fig. 5 shows that the cardboard and silica gels moxibustion device have higher combustion stability than the moxa sticks. The combustion stability of moxa sticks in the wooden and ceramic moxibustion devices is the worst among the four types of moxibustion devices: the larger the relative standard deviation of the infrared radiation intensity of the moxibustion apparatus working for 20 min, the smaller the stability, the greater the change of the infrared radiation intensity of the moxibustion apparatus working for 20 min, and the more unstable the infrared radiation intensity emitted by the moxibustion apparatus during the operation on the human body.

*vs control without device moxa sticks, #vs ceramic moxibustion device, Δvs wooden moxibustion device, MD means moxibustion device.

### 3.3 The infrared radiation of moxibustion devices at different times

In a TCM clinic, the usage time of moxibustion devices is about 20 min. We collected the infrared radiation spectrum every 2 min, for 20 min in total, and each detection took 49 s. The average intensities of infrared radiation spectrum at different times were recorded and analyzed.

**Fig. 6:**
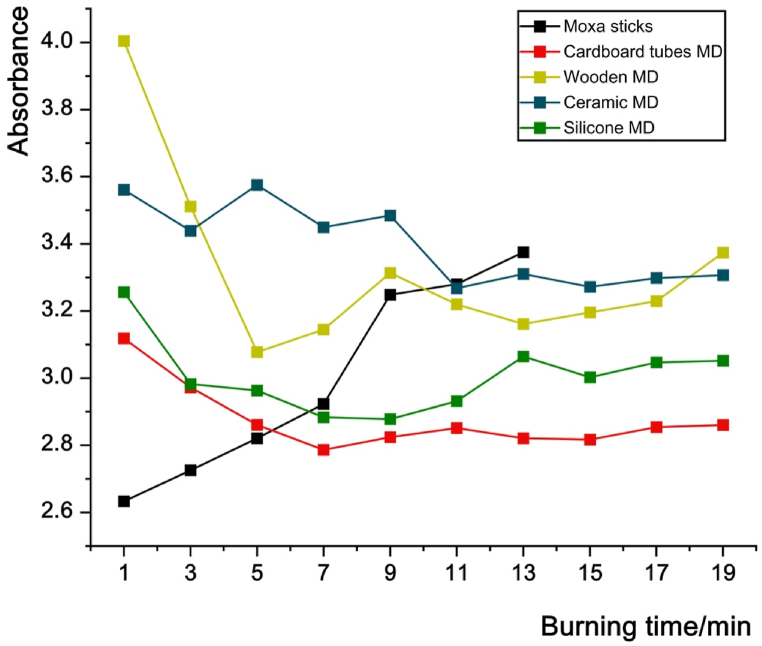
Infrared radiation intensity of the moxa sticks burned in moxibustion devices for different combustion times.

Fig. 6 shows the average value of absorbance and the change line of infrared radiation intensity. In the control group, the infrared radiation intensity of the moxa stick without the moxibustion device reached its maximum after ignition and then gradually decreased. Moxa sticks of the same length burned out at the 15th minute, and the infrared radiation was not collected after 15th minute.

In the 1st minute, the infrared radiation intensity of wooden moxibustion devices is the lowest in the 4 types of moxibustion devices. The infrared radiation intensity of the wooden moxibustion device increases rapidly, and reaches maximum in the 5th minute. Then the intensity decreases at 9th minute, and becomes unstable from 9th to 20th minutes. The infrared radiation intensity of ceramic moxibustion devices fluctuates repeatedly in the first 9 min and stabilizes gradually after the 11th minute, but the overall intensity is low in the ceramic moxibustion devices. The infrared radiation intensity of silicone moxibustion devices increases and stabilizes rapidly at the initial stage, but it weakens in second half of the combustion process. The cardboard moxibustion devices appear to perform best, with a stable and increased infrared radiation intensity which is maintained for a longer time.

**Fig. 7:**
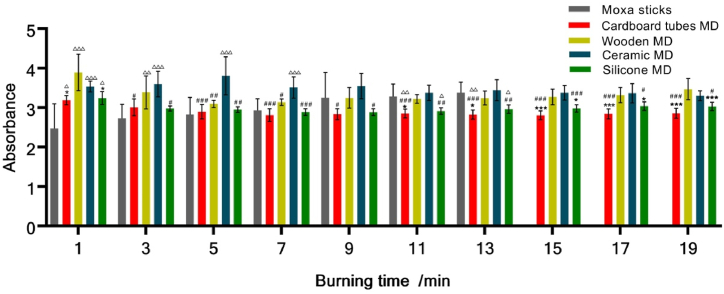
Repetitive analysis of infrared radiation intensity of moxibustion devices at different combustion times. *vs wooden moxibustion device, #vs ceramic moxibustion device, &vs control moxa sticks without device, MD means moxibustion device.

Fig. 7 shows the comparison of infrared radiation intensity from the 1st to the 19th minutes. In the 1st minute after ignition, the absorbance of the moxa stick without the moxibustion devices is significantly lower than with moxibustion devices, which demonstrates that the devices reduce the infrared intensity after initial ignition, but the wooden moxibustion device exhibits a faster rate of infrared radiation enhancement. In the 3rd minute, the cardboard and silica gels moxibustion devices are heated up gradually and the infrared radiation is strengthened. From 5th to 7th minute, the moxibustion devices made from cardboard tubes, wood and silica gels tend to have similar infrared radiation intensities; however, the ceramic moxibustion device heats up slowly and produces lower infrared radiation. Moxibustion devices can steadily increase the burning temperature of moxa sticks, and the cardboard and silica gels moxibustion devices have good performance from the 9th minute. Due to the fast burning rate of the moxa stick without a moxibustion device, it is extinct in the 15th minute. This also indicates that the moxibustion devices prolong the burning time of moxa sticks. Within 20 min of moxibustion, the infrared radiation of cardboard and silica gels moxibustion devices is strong and stable integrally; of the two, the cardboard moxibustion device is superior.

### 3.4 The intensity of FTIR at different wavebands

In this study, we collected infrared radiation with the wavelength range 1.28–25 μm, and further analyzed radiation intensity at 1.28–2.5 μm, 2.5–25 μm, and 7.5–10 μm.

**Fig. 8:**
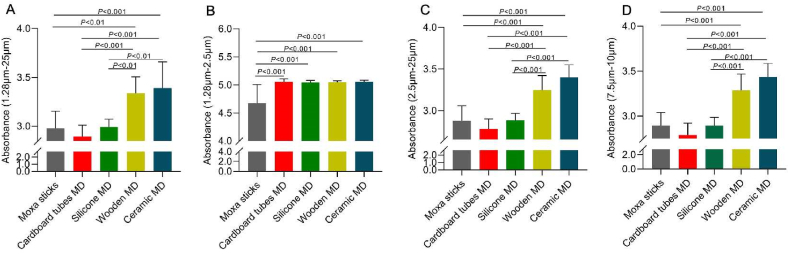
The infrared radiation intensity of FTIR at different wavebands A)1.28–25 μm, B) 1.28–2.5 μm, C) 2.5–25 μm, d)7.5–10 μm, MD means moxibustion device.

Fig. 8A shows the infrared radiation intensity in the 1.28–25 μm wavebands. The absorbance in cardboard and silica gels moxibustion devices made by are the closest to the burning of moxa sticks, and significantly lower than in the other two types of moxibustion devices. In the near infrared wavebands (1.28–2.5 μm, Fig. 8B), the intensity of moxa sticks is clearly stronger than that of moxibustion devices, which suggests that explains the moxibustion devices may absorb part of infrared radiation from the burning moxa sticks. In the 2.5–25 μm and 7.5–10 μm wavebands (Fig. 8C and D), there is not much difference between the burning of moxa sticks alone and in the cardboard and silica gel moxibustion devices.

### 3.5 Moxibustion devices of principal component analysis (PCA) score

**Fig. 9:**
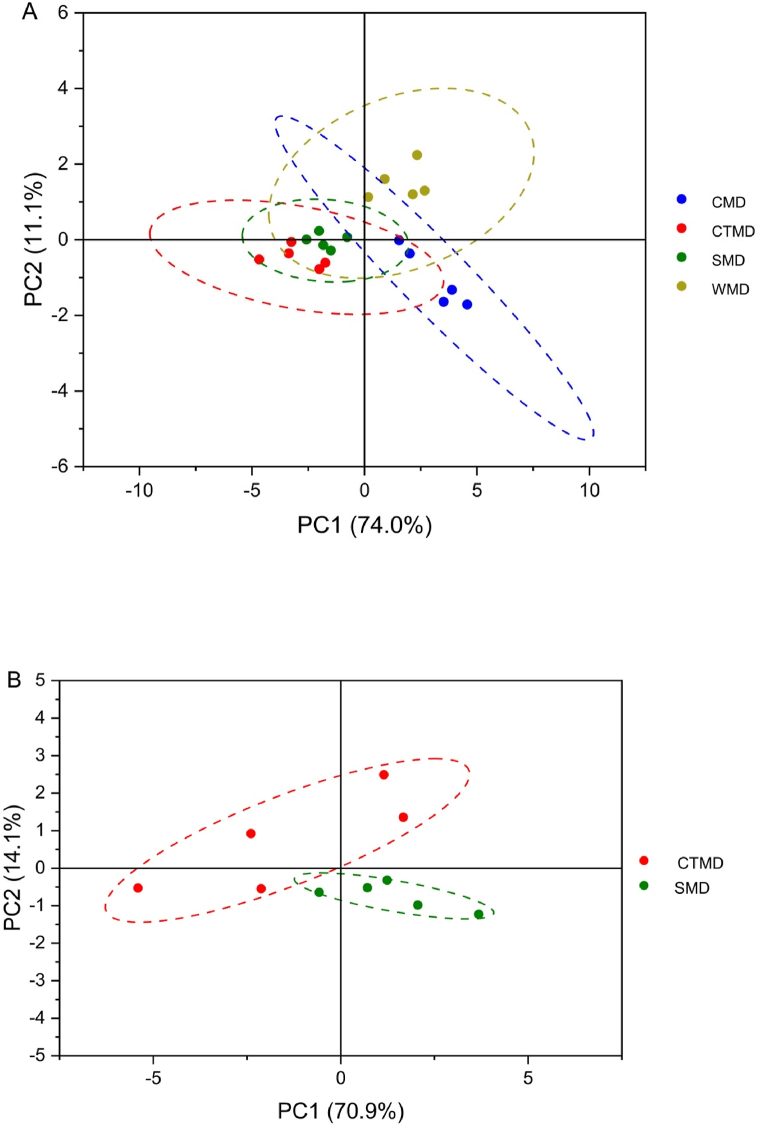
PCA score plot of infrared radiation intensity of moxibustion devices at different time points A) four moxibustion devices, B) moxibustion devices made from cardboard tubes vs silica gels.

In the PCA score plot, samples with the same infrared radiation intensity will cluster together, showing the clustering effect of each group of samples. Fig. 9A shows the infrared radiation intensity scores of four different moxibustion devices at different time points in the 1.28–25 μm wavelength range, and Fig. 9A and B shows that the four groups exhibit a certain degree of clustering. There are differences in the infrared radiation intensity of the four types of moxibustion devices at different time points.

**Fig. 10:**
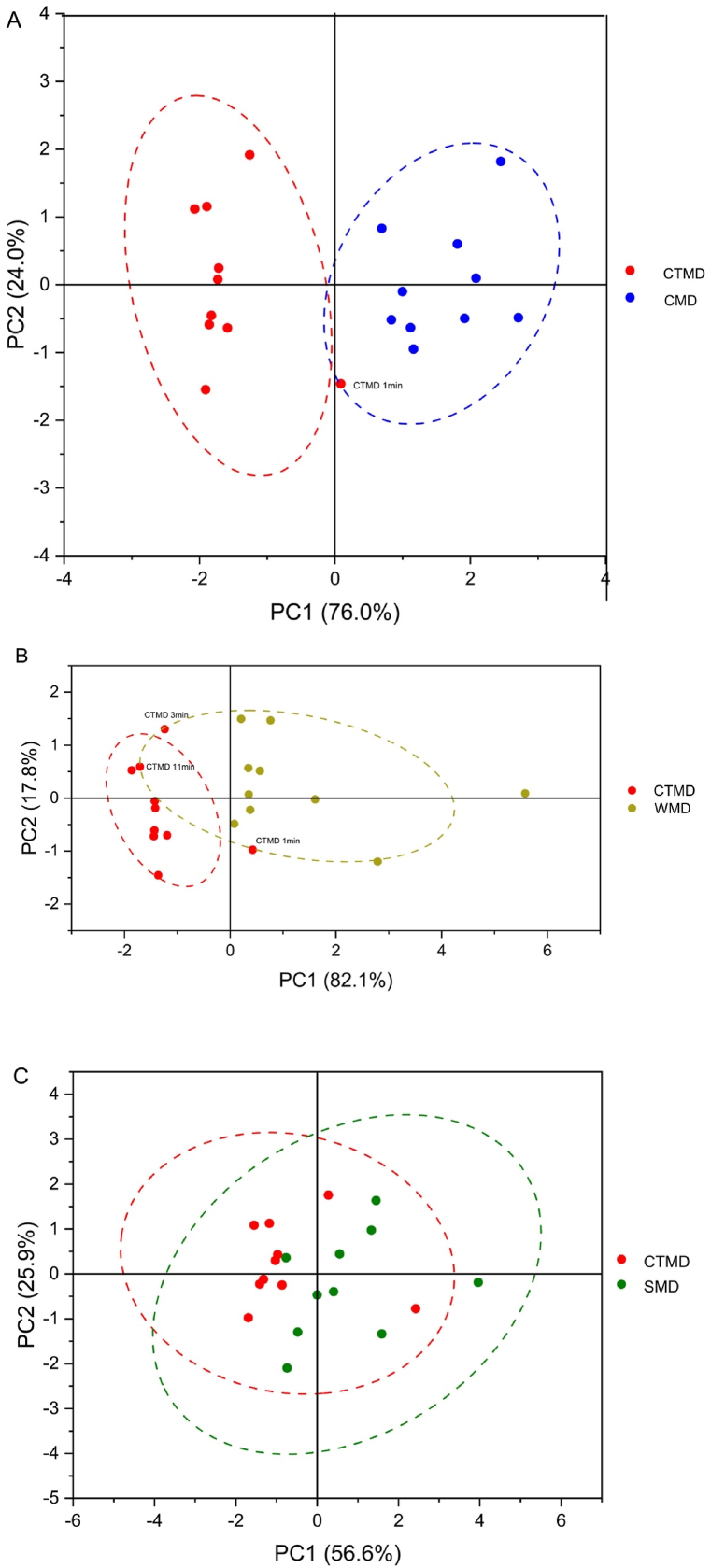
PCA score plot of infrared radiation intensity of moxibustion devices at different wavebands. A) cardboard moxibustion devices vs ceramics; B) cardboard moxibustion devices vs wood; C) cardboard moxibustion devices vs silica gels.

Having compared the cardboard moxibustion devices with the other three types of moxibustion devices, we have obtained PCA scores for infrared radiation intensity in different bands, which are 1.28–25 μm, 1.28–2.5 μm, 2.5–25 μm, and 7.5–10 μm. Fig. 10A shows a high similarity between the infrared radiation intensity of the first minute of cardboard moxibustion devices and the infrared radiation intensity of ceramic moxibustion devices from 1 to 20 min, which is the lowest infrared radiation among the whole burning period of cardboard moxibustion devices. Fig. 10B shows that the infrared radiation from cardboard moxibustion devices at 1st, 3rd, 11th minute has the similar effects to that from the ceramic moxibustion device in the 20 min of combustion. Fig. 10C illustrates the PCA score and Cluster degree of cardboard and silica gel moxibustion devices. Based on the PCA analysis, the two factors with the highest contribution are 1.28–2.5 μm and 2.5–25 μm. The differentiation between cardboard and silica gel moxibustion devices is not significant in the 1.28–2.5 μm and 2.5–25 μm wavebands.

## 4 Discussion

In ancient times, moxibustion was the earliest physical therapy method [22,23]. Infrared radiation of moxibustion is one of the important effective factors of moxibustion, which acts on the acupoints on human body [24]. The invisible infrared light emitted by the burning of moxa in the moxibustion device accounts for the majority of its radiation spectrum. There is radiation of varying intensities from red light to far infrared. As a direct and rapid collection technique, FTIR spectroscopy can profile the radiation intensity of moxibustion devices made of different materials.

The influence site of infrared radiation of moxibustion includes epidermal tissue and subcutaneous tissue. In this study, the infrared radiation transdermal ability of moxibustion devices was measured by comparing the infrared radiation of moxibustion devices covering simulated skin and without simulated skin. The key issue was whether the moxibustion warmer can assist the infrared radiation of moxibustion to reach the human body and thus play a warming effect. The results of this study show that the infrared radiation of the cardboard moxibustion device has the best ability to simulate the skin, and it is speculated that it has a good ability to assist the infrared radiation of moxa pillar to penetrate the superficial part of the human body in the process of clinical treatment, so as to reduce the loss of infrared radiation in vitro and the superficial part of the human body.

The results of this study showed that, compared with moxa sticks, the four kinds of moxibustion devices had stronger stability within 20 min, which was speculated to be due to the effect of the outer structure of moxibustion apparatus on the accumulation and accumulation of infrared radiation emitted by moxibustion devices, accompanied by the conversion of light and heat energy, reducing energy loss and ensuring the stability of infrared radiation intensity during the operation of moxibustion devices. Therefore, moxibustion devices can help improve the stability of infrared radiation intensity during moxibustion, and cardboard and silicone devices have the best stability. Due to the different thermal conductivity and heating rate of different materials of moxibustion devices, there are differences in the stability of infrared radiation intensity during the burning process of moxa sticks in different material moxibustion devices. Stable combustion can ensure the quality of moxibustion, thereby exerting its therapeutic effect.

According to the change trend of infrared radiation intensity of moxa sticks and each moxibustion devices within 20 min, the infrared radiation intensity of moxibustion devices showed a downward trend from the first minute, while the infrared radiation of devices increased with the extension of internal burning time. The reason for this phenomenon may be that part of the visible light emitted by the combustion of moxa sticks cannot be converted into heat energy or because the infrared radiation may be absorbed by substances in the air, resulting in the loss of visible light energy and infrared radiation, so the light energy generated by the combustion of moxa sticks cannot be maximized and accumulated in the human skin. Conversely, due to the effect of its external structure, part of the light energy generated by the combustion of moxa sticks inside the moxibustion devices can be converted into heat energy through the absorption of the external structure of the moxibustion devices, slowing down the loss of light energy generated by the combustion of moxibustion devices, so as to be used by the human body through heat transfer. The structure of moxibustion tube slows down the loss of heat energy generated by burning moxa sticks to a certain extent, so the moxibustion devices can reduce the loss of infrared radiation from burning moxa sticks in many ways.

In the field of medicine, the therapeutic effects may vary depending on the infrared wavelength range. Near infrared radiation has strong penetration ability wavelength range, and mid to far infrared radiation can generate thermal effects [[25], [26], [27], [28]], The absorbance in 2.5–25 μm wavebands reflects the intensity of mid to far infrared radiation, which is related to thermal effects. The wavebands in 7.5–10 μm reveal the capacity of the human body to absorb infrared radiation [29,30]. The above results indicate that two cardboard and silica gel moxibustion devices exhibit good performance in generating thermal effects and human absorption and utilization. In this experimental study, it was found that in the process of moxibustion application of four kinds of moxibustion devices, the external structure of ceramic moxibustion devices had the highest temperature. Combined with the results of this experiment, it was speculated that the reason for its weak infrared radiation might be due to the excessive absorption of energy emitted by moxa sticks combustion by its external structure, which led to the preferential absorption of heat energy or light energy by the external structure. As a result, the energy loss of the ceramic moxibustion devices were more than that of the other moxibustion devices.

In clinical treatment, there are many kinds of moxibustion devices to choose, and it is very important to choose the better material moxibustion device. In this study, the infrared radiation from cardboard moxibustion devices at the 1st, 3rd, and 11th minutes has the similar effects to the ceramic moxibustion device in the 20 min of combustion. We have observed that whatever the material of the moxibustion devices are, the overall trend of the intensity radiation of the moxibustion devices are upward. It indicates that although the ceramic moxibustion devices accumulates enough infrared radiation in 20 min, it is still only comparable to cardboard moxibustion devices’ infrared radiation in minutes 1, 3, and 11. Therefore, in clinical treatment, the therapeutic effect of cardboard moxibustion devices may be better than that of ceramic moxibustion devices made by ceramic.

## 5 Conclusion

This study is based on the Fourier transform infrared spectrometer to explore the infrared radiation spectral properties of moxibustion devices. Starting from the spectral shape, infrared radiation intensity, and multiple infrared spectral wavebands, the infrared spectral characteristics of moxibustion devices were comprehensively studied.

In total, moxibustion devices can prolong the burning time of moxa sticks, allowing the warm stimulation of moxa sticks to continue to affect the human body. The cardboard and silica gel moxibustion devices perform better with respect to Combustion stability and infrared radiation intensity. PCA analysis indicates there is difference between the moxibustion devices made by cardboard tubes and silica gels. The cardboard moxibustion devices made by cardboard tubes can stabilize and increase the intensity of emitted infrared radiation within 20 min of moxibustion. Therefore, cardboard tubes and silicone are the best materials for making moxibustion devices, with cardboard tubes being superior.

## Data availability statement

The original contributions presented in the study are included in the article/Supplementary Material, further inquiries can be directed to the corresponding author/s.

## CRediT authorship contribution statement

**Jiachen Zhang:** Writing – original draft, Software, Investigation. **Jing He:** Writing – original draft, Software, Investigation. **Shuang Shuang:** Methodology, Investigation. **Yuqing Shi:** Visualization, Investigation. **Li Han:** Methodology, Investigation. **Xin Hui:** Visualization, Methodology. **Xiali Ouyang:** Methodology, Investigation. **Jingyi Zhu:** Methodology, Investigation. **Zhongyu Wang:** Validation, Investigation. **Baixiao Zhao:** Writing – review & editing, Conceptualization. **Rui He:** Writing – review & editing, Resources, Conceptualization.

## Declaration of competing interest

The authors declare the following financial interests/personal relationships which may be considered as potential competing interests: Rui He and Beixiao Zhao reports financial support was provided by 10.13039/501100004846Beijing University of Chinese Medicine.This work was supported by 10.13039/501100012166National Key Research and Development Program (No. 2019YFC1711904). If there are other authors, they declare that they have no known competing interests or personal relationships that could have appeared to influence the work reported in this paper.
